# The association between physical activity and mobile phone dependence in college students: serial indirect associations through psychological capital and self-control

**DOI:** 10.3389/fpsyg.2026.1911519

**Published:** 2026-10-21

**Authors:** Wenwen Pan, Yong Zhang, Tao Shan

**Affiliations:** 1 School of Physical Education, Qiqihar University, Qiqihar, Heilongjiang, China; 2 School of Physical Education, Liaoning Finance and Trade University, Xingcheng, Liaoning, China

**Keywords:** cross-sectional survey, mobile phone dependence, physical activity, psychological capital, self-control, serial indirect association

## Abstract

**Background:**

Mobile phone dependence is receiving increasing attention in university populations. Existing evidence suggests that physical activity is related to psychological wellbeing and self-regulatory processes; however, the pathways that may account for its association with mobile phone dependence remain unclear. The present study explored the relationship between physical activity and mobile phone dependence by examining psychological capital and self-control as sequential indirect factors in the proposed model.

**Methods:**

Using a cross-sectional design, we surveyed a convenience sample of 567 students from four universities in China. Participants completed self-report measures of physical activity, psychological capital, self-control, and mobile phone dependence. We computed Pearson correlations and applied PROCESS model 6 with 5,000 bootstrap resamples, adjusting the model for gender, age, and grade.

**Results:**

Physical activity showed an inverse correlation with mobile phone dependence (*r* = −0.246, *p* < 0.001). A significant overall association was also observed between PA and MPD (*β* = −0.255, *p* < 0.001). However, after psychological capital, self-control, and the covariates were included in the model, the remaining direct coefficient was no longer significant (*β* = −0.043, *p* = 0.265). Bootstrap estimates supported three indirect pathways: via psychological capital (−0.069, 95% CI [−0.104, −0.035]), via self-control (−0.108, 95% CI [−0.157, −0.060]), and sequentially through psychological capital followed by self-control (−0.036, 95% CI [−0.060, −0.014]).

**Conclusion:**

Overall, the results suggest that the PA–MPD relationship was primarily reflected in indirect associations involving psychological capital and self-control within the proposed cross-sectional model. The findings underscore the relevance of PsyCap and SC to the association between physical activity and mobile phone dependence among college students.

## 1 Introduction

Due to their ubiquitous use, mobile phones have become essential for socializing, entertainment, work, and education (Mahapatra, 2019). The risks associated with excessive mobile phone use have received increasing scholarly attention as a result of this global trend. Evidence from an expanding international literature suggests that poorer cognitive functioning, weaker self-regulatory capacity, and elevated levels of emotional instability are correlated with excessive mobile phone use (Billieux, 2012; Kuss and Griffiths, 2017; Soraci et al., 2025). Furthermore, in a range of cultural contexts, meta-analytic findings demonstrate that excessive mobile phone use is consistently linked to stress, anxiety, and depression (Lazo-Caparrós et al., 2025; Zhong et al., 2022). Mobile phone dependence (MPD), defined as excessive reliance on mobile phones accompanied by withdrawal, lack of control, and functional dysfunction (Nikolic et al., 2023), has become a global concern among young adults. In China, recent evidence indicates that 38.6% of college students experience symptoms of MPD (Lin et al., 2022). Elevated MPD levels have been linked to worse academic performance, worse social functioning, and decreased general wellbeing among college students (Acikgoz et al., 2022; Zhang and Zeng, 2024), making it important to identify its key correlates and associated psychological processes.

Current research on MPD among college students primarily focuses on two areas. The first direction concerns factors associated with MPD. Research in this field has looked at individual traits like physical activity (PA) (Lu et al., 2022; Yang et al., 2021) and psychological capital (PsyCap) (Wang et al., 2021), as well as social and environmental variables like peer pressure (Huang et al., 2021), parenting and family factors (Doo and Kim, 2022), and different types of perceived stress (Wang et al., 2021). Additional experimental findings indicate that self-reported mobile phone craving was lower among college students with MPD after a short workout of moderate-level aerobic exercise than among participants in the control condition (Yang et al., 2022). Recent studies have further situated PA within more complex models: PA moderated associations involving loneliness, social anxiety, and MPD (Wang et al., 2025; Wang J. et al., 2024a), while another chain-mediation model linked PA, stress, MPD, and sleep quality (Wang J. F. et al., 2024). The second direction focuses on outcomes associated with excessive mobile phone use. Reported correlates of MPD include academic burnout, social anxiety, elevated stress, insomnia, and other adverse emotional outcomes (Abu Khait et al., 2025; Chen et al., 2021).

This study addresses an integrative rather than an evidentiary gap. Previous studies have examined indirect associations involving PsyCap and SC separately in the relationship between PA and MPD (Chen et al., 2022; Zhong et al., 2021), while related pathways involving self-esteem and subjective wellbeing have also been reported (Ke et al., 2024; Wang J. et al., 2024b). Building on these findings, examining PsyCap and SC within the same model might offer a greater integrated comprehension of the mental procedures associated with the PA–MPD relationship. In particular, it remains unclear whether PsyCap and SC are associated with MPD through distinct indirect pathways, a serial pathway, or both. PsyCap reflects broader motivational and emotional resources, such as self-efficacy, hope, optimism, and resilience, whereas SC reflects a more proximal capacity for impulse inhibition and behavioral control. From a self-regulation perspective, these broader psychological resources may support sustained goal pursuit and behavioral regulation, making SC a theoretically plausible more proximal correlate following PsyCap (Xu et al., 2025). We therefore assessed the PA–MPD association among college students, alongside independent and serial indirect associations involving PsyCap and SC.

## 2 Theoretical background and hypotheses

### 2.1 Physical activity and Mobile phone dependence

PA has increasingly been examined as a behavioral correlate of MPD. Activities such as running, yoga, fitness training, and organized competitive sports not only involve bodily movement but also provide opportunities for goal pursuit, enjoyment, and offline social interaction (Morgan and Ellickson, 2021). MPD may also conflict with time spent on these activities. These traits offer a foundation for investigating the connection between MPD and PA. Previous studies have reached inconsistent conclusions regarding the PA–MPD association. Some studies report no clear association between PA frequency and MPD among college students (Barkley and Lepp, 2016), whereas others have identified an inverse association between PA and MPD (Lu et al., 2022; Yang et al., 2021). Additionally, evidence has revealed that college students with MPD who participated in an acute session of moderate-level aerobic activity reported less need for their phones than those in the control condition (Yang et al., 2022). These differences may reflect variations in samples, measurement approaches, research settings, and the indicators used to assess PA, such as frequency, intensity, and duration. They might also indicate a lack of focus on the psychological mechanisms that underlie the connection between PA and MPD. Most previous studies have treated PA primarily as an external behavioral correlate and have paid less attention to how internal psychological resources and self-regulatory capacities may be involved in this relationship. Drawing on the prevailing empirical findings, we hypothesize:

*H1:* PA is significantly negatively correlated with MPD.

### 2.2 The indirect role of psychological capital

PsyCap, which comprises self-efficacy, optimism, hope, and resilience, is a key positive psychological resource associated with coping with stress and pursuing important goals (Luthans and Youssef, 2004). The potential positive association between PA and PsyCap may be interpreted in light of mastery experiences, opportunities for goal attainment, positive emotions, and experiences of coping with difficulty that can accompany exercise (Acar, 2022; Zhang et al., 2024). Specifically, a study including 519 college students discovered a favorable correlation between exercise and PsyCap (Liu M. et al., 2022). Additionally, studies have demonstrated a negative correlation between PsyCap and MPD (Simsek and Sali, 2014; Sun et al., 2022; Zhang et al., 2021). Among college students, higher PsyCap may be linked to more adaptive psychological resources for managing stress and negative emotions, alongside a lower likelihood of excessive reliance on mobile phones for escape, reassurance, or mood regulation. Chen et al. (2022) further identified a statistically significant indirect association between PA and MPD through PsyCap. Evidence involving related positive psychological resources also supports the plausibility of this indirect association. Ke et al. (2024) reported a significant indirect association between PA and smartphone addiction involving self-esteem, while Lu et al. (2022) and Wang J. et al. (2024b) reported indirect associations involving subjective wellbeing in related models of PA, MPD, and online addiction. Taken together, these results demonstrate that PsyCap could indicate a key psychological link between PA and MPD. PA may be positively associated with the psychological resources embodied in PsyCap, whereas the negative association between PsyCap and MPD may reflect more adaptive coping with stress and negative emotions. The present study therefore examines whether PsyCap independently accounts for part of the association between PA and MPD. This reasoning leads to the following hypothesis:

*H2:* PA is indirectly and negatively associated with MPD through PsyCap.

### 2.3 The indirect role of self-control

The ability to control one’s thoughts, feelings, and actions in a way that promotes goal-directed action and the pursuit of long-term objectives is referred to as SC (Tangney et al., 2004). This ability involves higher-order cognitive processes related to behavioral regulation and impulse control. Evidence indicates that PA is positively associated with SC (Potoczny et al., 2025; Zhou et al., 2023). Prefrontal functions involved in executive control may be relevant to this association (Moore et al., 2022; Pan et al., 2023). Cheng and Wang (2020) found that college students who were better able to regulate their actions or thoughts during PA were more likely to complete tasks within a specified timeframe and reported higher levels of SC. At the same time, SC is an important psychological correlate of MPD. Individuals with higher SC may report a greater capacity to resist smartphone-related urges and lower levels of excessive mobile phone use (Chen and Zhang, 2024; Liu F. B. et al., 2022; Sok et al., 2019). Yang et al. (2019) and Zhong et al. (2021) further reported significant indirect associations between PA and MPD involving SC. However, the role of SC in the association between PA and MPD warrants further examination. This reasoning leads to the following hypothesis:

*H3:* PA is indirectly and negatively associated with MPD through SC.

### 2.4 The serial indirect roles of psychological capital and self-control

The self-regulation perspective provides a relevant basis for integrating PsyCap and SC (Carver and Scheier, 1998). From this perspective, effective behavioral regulation is associated not only with the capacity to inhibit impulses but also with broader motivational and emotional resources that support goal pursuit. PA-related experiences involving mastery, goal pursuit, positive feedback, and coping with challenges have been associated with higher PsyCap (Wei et al., 2024). The theoretically specified PsyCap → SC ordering was selected based on the different functional positions of the two constructs in relation to MPD. Hope, optimism, self-efficacy, and resilience are just a few of the positive psychological resources that PsyCap stands for (Wei et al., 2024). These resources are relevant to sustained goal pursuit, coping with difficulties, and the maintenance of self-regulation (Liu et al., 2020; Morrison and Pidgeon, 2017; Oriol et al., 2020). In contrast, SC reflects a more proximal regulatory capacity in specific behavioral contexts, including resisting mobile phone-related temptations, inhibiting habitual checking, and maintaining goal-directed behavior (Chen and Zhang, 2024; Liu F. B. et al., 2022). Accordingly, within the present PA–MPD framework, PsyCap is conceptualized as a broader psychological resource associated with self-regulation, whereas SC is viewed as a more proximal regulatory capacity relevant to the management of mobile phone use. A similar ordering has also been examined in prior serial indirect-association research (Xu et al., 2025). Prior research has investigated indirect associations between PA and mobile phone–related outcomes, assessing PsyCap and SC separately (Chen et al., 2022; Zhong et al., 2021). Examining them simultaneously allows their independent indirect associations and the theoretically specified serial association to be tested within the same model. Drawing on the prevailing empirical findings, we hypothesize:

*H4:* PA is indirectly and negatively associated with MPD through a serial pathway involving PsyCap followed by SC.

Guided by the preceding theoretical rationale, we assessed the PA → MPD association among college students, alongside independent and serial indirect associations involving PsyCap and SC. Figure 1 illustrates the hypothesized model.

**Figure 1:**
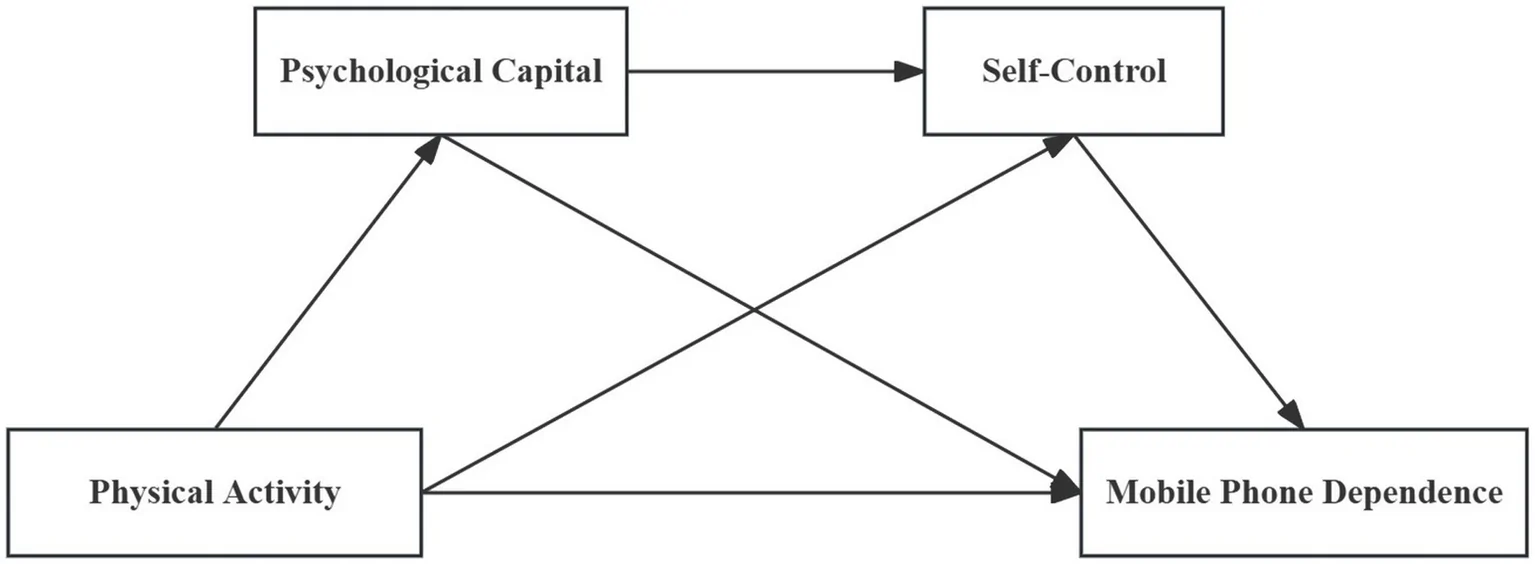
Hypothesized research model.

## 3 Materials and methods

An online cross-sectional survey was conducted from May to June 2026 among students from four comprehensive undergraduate universities in Northeast China: Qiqihar University and Jiamusi University in Heilongjiang Province, Beihua University in Jilin Province, and Dalian University in Liaoning Province. Because recruitment depended on access to cooperating classes, convenience sampling was employed. The research team coordinated with instructors from different academic departments, who introduced the study and distributed the survey link through the relevant class groups. Eligible students voluntarily decided whether to participate. While informed consent was acquired from their legal guardians, participants under the age of 18 gave their own assent. Those aged 18 years or above gave informed consent on their own behalf. All consent and assent requirements were fulfilled before participants proceeded to complete the questionnaire. To protect participant privacy, the questionnaire did not record university affiliation at the individual level; therefore, university-specific sample sizes could not be reported. Class identifiers and the total number of participating classes were also not retained in the analytic dataset. Consequently, potential clustering within classes or universities could not be evaluated. Ethical approval was obtained from the Ethics Committee of Qiqihar University (Approval Number: QDTY-01).

We used G*Power 3.1.9.7 to determine the sample size *a priori* for fixed-model multiple regression with *R*^2^ departure from zero (Faul et al., 2009). Assuming *f*^2^ = 0.05, *α* = 0.05, power = 0.95, and six predictors, namely PA, PsyCap, SC, gender, age, and grade, in the regression equation predicting MPD, the minimum sample size was 424. Because the primary analysis examined indirect associations, the mediation-specific benchmark proposed by Fritz and MacKinnon (2007) was also considered; under conservative assumptions, at least 558 participants were required for 0.80 power in a percentile-bootstrap mediation test. Of 596 questionnaires collected, 29 were excluded because they were incomplete, completed in an implausibly short time, or showed patterned responding, such as repeatedly selecting the same response option across items, leaving 567 valid responses (95.13%). Participants represented different academic disciplines. Eligibility also required regular participation in PA for at least one complete academic semester. This criterion was used to ensure that participants had sufficient prior PA experience to respond meaningfully to the PA measure. Sophomores comprised 65.96% of the sample, followed by freshmen (26.10%), juniors (4.59%), and seniors (3.35%). Table 1 displays all of the demographic information.

**Table 1:** Sample demographic profile.

Demographic characteristics	Category	Quantity	Percentage (%)
Gender	Male	346	61.02
	Female	221	38.98
Age	18 years old or younger	122	21.52
	19–22 years old	439	77.42
	23 years and above	6	1.06
Grade	Freshman	148	26.10
	Sophomore	374	65.96
	Junior	26	4.59
	Senior	19	3.35

*N* = 567.

### 3.1 Physical self-description questionnaire (PSDQ)

The present study used an existing Chinese-language version of the six-item Activity subscale adapted from the PSDQ to assess self-perceived participation in PA among college students (Marsh et al., 1994; Yang, 2002). Representative items include “I exercise at least three times per week (e.g., jogging, dancing, or playing basketball)” and “I participate in many physical activities, such as dancing, swimming, and ball games.” No specific recall period was specified; participants responded according to their usual physical activity patterns. Items were rated on a 5-point Likert scale, with no reverse-scored items, and higher scores indicated greater self-perceived participation in PA. Bartlett’s test of sphericity was significant (*p* < 0.001), KMO = 0.784, and Cronbach’s α was 0.783. The single-factor CFA showed good model fit, *χ*^2^_(9)_ = 13.480, *χ*^2^/df = 1.498, CFI = 0.999, TLI = 0.998, RMSEA = 0.030 (95% CI [0.000, 0.060]), and SRMR = 0.015. The range of standardized factor loadings was 0.731–0.782.

### 3.2 Positive psychological capital questionnaire (PPQ)

The PPQ created by Zhang (2010) served as the basis for the PsyCap Scale employed in this investigation. The PPQ was chosen because it comprehensively reflects the four key components of PsyCap (self-efficacy, optimism, hope, and resilience), all of which are theoretically linked to the associations examined in this study (Li et al., 2024). A higher degree of PsyCap is indicated by higher scores on the 26 items on the scale, which are scored on a 7-point Likert scale. High reliability was shown by the scale (Cronbach’s *α* = 0.923, KMO = 0.969, Bartlett’s test *p* < 0.001). Because PsyCap was analyzed as an overall construct in the subsequent analyses, a single-factor CFA was used to evaluate the measurement structure of the total PsyCap score. The model showed good overall fit, *χ*^2^_(299)_ = 313.432, *χ*^2^/df = 1.048, CFI = 0.996, TLI = 0.996, RMSEA = 0.009, and SRMR = 0.026. Standardized factor loadings ranged from 0.501 to 0.646.

### 3.3 Self-control scale (SCS)

Self-control (SC) was assessed using the 13-item Brief Self-Control Scale (Tangney et al., 2004), a commonly applied measure of general self-regulatory capacity in behavioral research (Li et al., 2024). Items were scored on a five-point scale, with nine negatively phrased items reverse-coded so that higher scores indicated stronger self-control. The scale showed satisfactory reliability in the present sample (Cronbach’s *α* = 0.881; McDonald’s *ω* = 0.881). Sampling adequacy was supported by a KMO value of 0.940, while Bartlett’s test was significant (*p* < 0.001). CFA further indicated an acceptable one-factor structure: *χ*^2^_(65)_ = 152.740, *χ*^2^/df = 2.350, CFI = 0.959, TLI = 0.951, RMSEA = 0.050 (95% CI [0.039, 0.060]), and SRMR = 0.036. The range of standardized factor loadings was 0.513–0.688.

### 3.4 Smartphone addiction scale (SAS)

MPD was assessed using five items adapted from the SAS (Mason et al., 2022). The instrument’s original name is retained solely to identify its source; in the present study, the resulting scores were interpreted as levels of MPD rather than as evidence of a clinical addiction diagnosis. The five items were graded using a 5-point Likert scale; higher scores indicated higher levels of MPD. Reliability analysis indicated strong reliability (Cronbach’s *α* = 0.820, KMO = 0.784, Bartlett’s test *p* < 0.001). The single-factor CFA showed good model fit, with *χ*^2^_(5)_ = 2.976, *χ*^2^/df = 0.595, *p* = 0.704, CFI = 1.000, TLI = 1.000, RMSEA = 0.000 (95% CI [0.000, 0.044]), and SRMR = 0.007. Standardized factor loadings ranged from 0.761 to 0.818.

### 3.5 Statistical analysis

Data analysis was performed using SPSS 27.0 and R. SPSS was used to obtain descriptive statistics and Pearson correlation coefficients, whereas measurement models were examined with the lavaan package in R. Because the questionnaire items were ordinal, CFA parameters were estimated with WLSMV. PA, SC, and MPD were each modeled as unidimensional constructs, while PsyCap was represented by four correlated factors. The models were tested as originally specified, without item removal, residual correlations, or data-driven modifications. Complete standardized loadings are reported in Supplementary Table S6. The adequacy of the measurement models was evaluated with *χ*^2^/df, CFI, TLI, RMSEA, and SRMR. Serial indirect effects were then estimated with PROCESS Model 6. Gender, age, and grade were entered as covariates in every regression equation because of their potential relevance to physical activity and mobile phone use. Bootstrap inference based on 5,000 resamples was used for indirect associations, with an effect considered significant when the corresponding 95% confidence interval did not contain zero. All analyses used the same sample of 567 participants. The predicted pathways were considered as associations rather than proof of causal linkages because the data were cross-sectional.

## 4 Results

### 4.1 Discriminant validity

The heterotrait–monotrait ratio of correlations (HTMT) criterion was applied to examine whether the constructs were empirically distinct from one another. The HTMT values were 0.515 for PA–PsyCap, 0.329 for PA–SC, 0.285 for PA–MPD, 0.332 for PsyCap–SC, 0.367 for PsyCap–MPD, and 0.721 for SC–MPD. Adequate discriminant validity among the four components was supported by all values falling below the conservative threshold of 0.85.

### 4.2 Common method Bias test

Potential common method bias (CMB) was evaluated through two complementary diagnostic procedures. First, an exploratory factor analysis without rotation showed that the dominant factor explained only 14.631% of the overall variance, well below a level that would indicate substantial variance attributable to a single common source (Podsakoff et al., 2003). We then conducted a more restrictive test by specifying one latent construct underlying all 50 observed items. This model provided an unsatisfactory representation of the data, *χ*^2^_(1175)_ = 3960.554, *χ*^2^/df = 3.371, CFI = 0.724, TLI = 0.712, RMSEA = 0.065 (95% CI [0.063, 0.067]), and SRMR = 0.083. Taken together, these diagnostic results provide no indication that a single common factor dominated the observed responses. Nevertheless, these procedures are diagnostic rather than definitive tests of CMB (Richardson et al., 2009). Since all constructs were assessed through self-report during the same survey session, shared method variance may still have some influence and cannot be totally ruled out (Podsakoff et al., 2003).

### 4.3 Collinearity test

Table 2‘s statistics demonstrate that there are no serious multicollinearity issues because all variables’ tolerance is larger than 0.1 and the VIF values are less than 3.3 (Hair et al., 2019).

**Table 2:** Collinearity test.

Variable	Tolerance	VIF
PA	0.792	1.263
PsyCap	0.803	1.246
SC	0.911	1.098

*N* = 567. PA = physical activity; PsyCap = psychological capital; SC = self-control; VIF = variance inflation factor.

### 4.4 Descriptive characteristics of the sample and correlation analyses

Descriptive results and bivariate correlations are reported in Table 3. All associations reached statistical significance and followed the hypothesized directions, warranting subsequent testing of the proposed indirect pathways.

**Table 3:** Descriptive statistics, standard deviations, and correlations among the study variables.

Variable	M	SD	PA	PsyCap	SC	MPD
PA	19.647	4.111	1			
PsyCap	116.714	13.425	0.424***	1		
SC	38.898	4.842	0.264***	0.239***	1	
MPD	14.582	3.365	−0.246***	−0.304***	−0.582***	1

*N* = 567; PA = physical activity; PsyCap = psychological capital; SC = self-control; MPD = mobile phone dependence; M = mean; SD = standard deviation; ***p < 0.001.

### 4.5 Serial indirect-association analysis

Results of the serial indirect-association analysis are provided in Table 4 and Figure 2. PA was positively related to PsyCap (*β* = 0.424, *p* < 0.001), while both PA (*β* = 0.203, *p* < 0.001) and PsyCap (*β* = 0.160, *p* < 0.001) showed positive coefficients for SC. In turn, PsyCap (*β* = −0.162, *p* < 0.001) and SC (*β* = −0.532, *p* < 0.001) were negatively associated with MPD. No significant direct association between PA and MPD was detected (*β* = −0.043, *p* = 0.265).

**Table 4:** Regression results for the serial indirect-association model.

Regression equation		Fitting index			Significance of coefficients		
Outcome variable	Predictor variable	*R*	*R^2^*	*F*	*β*	*t*	*p*
PsyCap	PA	0.427	0.182	31.326***	0.424	10.834	<0.001
SC	PA	0.310	0.096	11.949***	0.203	4.478	<0.001
	PsyCap				0.160	3.599	<0.001
MPD	PA	0.608	0.370	54.775***	−0.043	−1.115	0.265
	PsyCap				−0.162	−4.307	<0.001
	SC				−0.532	−15.063	<0.001

*N* = 567. PA = physical activity; PsyCap = psychological capital; SC = self-control; MPD = mobile phone dependence; *R* = multiple correlation coefficient, *R*^2^ = coefficient of determination, *F* = model F statistic, *β* = standardized regression coefficient, *t* = t statistic, and *p* = probability value. ***p < 0.001.

**Figure 2:**
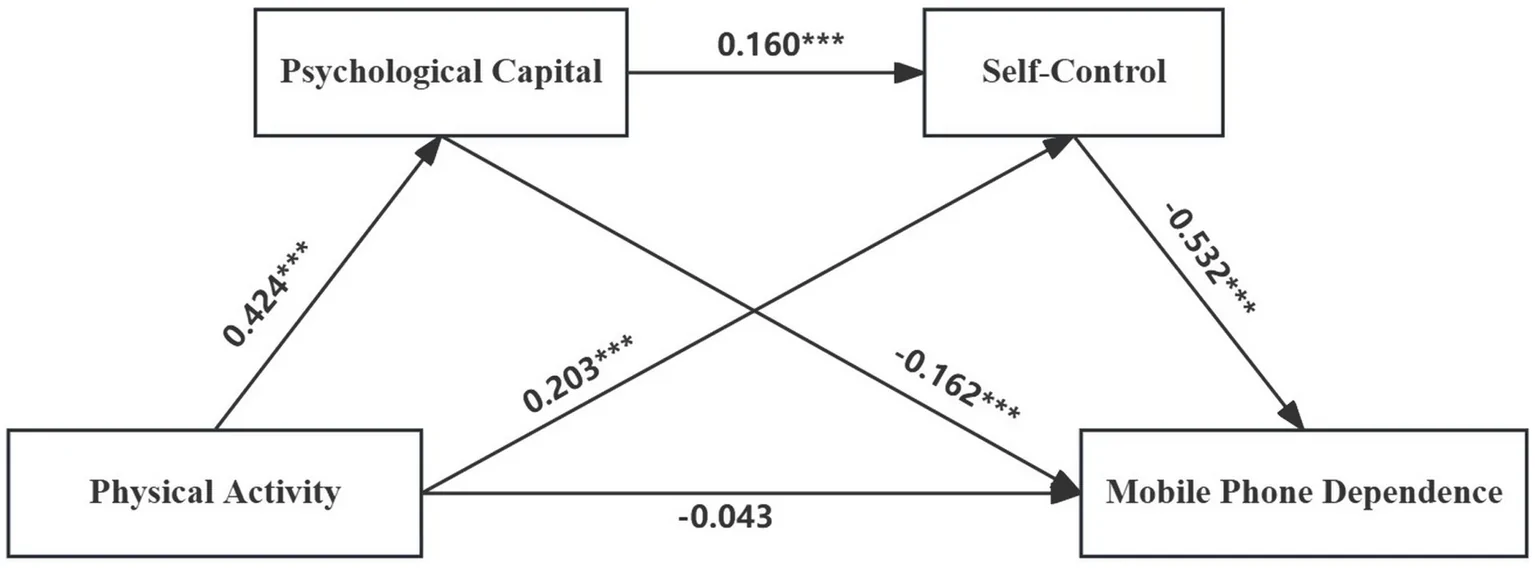
Serial indirect-association model. ***p < 0.001.

Bootstrap estimation identified three significant indirect PA–MPD associations (Table 5). The overall association between PA and MPD was negative and statistically significant (*β* = −0.255, *p* < 0.001). After PsyCap, SC, and the covariates were entered into the model, the remaining direct coefficient was small and nonsignificant (*β* = −0.043, *p* = 0.265). The combined indirect estimate was −0.212 (Boot SE = 0.030, 95% CI [−0.271, −0.154]), corresponding descriptively to 83.198% of the total association. The specific indirect estimate through PsyCap was −0.069 (Boot SE = 0.017, 95% CI [−0.104, −0.035]), while the estimate through SC was −0.108 (Boot SE = 0.025, 95% CI [−0.157, −0.060]). The sequential pathway involving both PsyCap and SC yielded an estimate of −0.036 (Boot SE = 0.012, 95% CI [−0.060, −0.014]). These three pathways represented 26.855, 42.221, and 14.122% of the total association, respectively. Because none of the corresponding bootstrap confidence intervals included zero, all three indirect associations were statistically significant. Of the specific indirect pathways, the SC pathway produced the largest absolute estimate.

**Table 5:** Total, direct, and indirect associations of PA with MPD.

Pathway	Standardized estimate	Boot SE	95% CI	Relative proportion (%)
Total association	−0.255***		[−0.337, −0.173]	
Direct association	−0.043		[−0.119, 0.033]	
Total indirect association	−0.212	0.030	[−0.271, −0.154]	83.198
PA → PsyCap → MPD	−0.069	0.017	[−0.104, −0.035]	26.855
PA → SC → MPD	−0.108	0.025	[−0.157, −0.060]	42.221
PA → PsyCap → SC → MPD	−0.036	0.012	[−0.060, −0.014]	14.122

*N* = 567. PA = physical activity; PsyCap = psychological capital; SC = self-control; MPD = mobile phone dependence; Indirect-association CIs were based on 5,000 bootstrap resamples. Relative proportion = indirect association/total association × 100%; proportions are descriptive. Full unstandardized and standardized estimates are reported in Supplementary Table S4. ***p < 0.001.

## 5 Discussion

### 5.1 Physical activity and Mobile phone dependence

In this college student sample, PA and MPD were significantly negatively correlated (*r* = −0.246, *p* < 0.001), supporting H1. The total PA–MPD association was also significant (*β* = −0.255, *p* < 0.001), whereas the direct PA–MPD association was not statistically significant after accounting for PsyCap, SC, and the covariates (*β* = −0.043, *p* = 0.265). This pattern does not prove a causal mediation mechanism, but it is compatible with an indirect-association structure including PsyCap and SC within the given cross-sectional model when combined with the substantial indirect relationships. The negative link is consistent with past studies that found decreased MPD among college students who participate in PA more regularly (Lu et al., 2022; Yang et al., 2021). Greater PA may be associated with more time spent in outdoor or sports-related activities and, correspondingly, less time available for smartphone use (Barkley and Lepp, 2016). Among students with MPD, experimental research has also linked a single bout of aerobic exercise to lower smartphone craving (Yang et al., 2022). In addition, the teamwork and face-to-face interaction involved in PA may be associated with lower loneliness and less reliance on virtual social platforms (Tong and Meng, 2023; Wang et al., 2025). These psychological and social factors may help contextualize the observed negative correlation between PA and MPD despite the nonsignificant direct association. Because PA was assessed using the PSDQ Activity subscale, this finding should be understood as an association involving self-perceived participation in PA rather than objectively measured activity frequency, intensity, duration, or energy expenditure.

### 5.2 The indirect roles of psychological capital and self-control

The indirect association between PA and MPD through PsyCap was significant (standardized indirect association = −0.069, 95% CI [−0.104, −0.035]), supporting H2. This result is in line with Chen et al. (2022), who found an indirect association between exercise and MPD involving PsyCap. Participation in PA may provide mastery experiences and supportive social environments associated with greater self-efficacy, optimism, hope, and resilience (Liu M. et al., 2022). More effective emotion regulation and lower reliance on smartphones for coping or distraction may be linked to these psychological resources. Greater PsyCap may also be associated with better behavioral regulation and greater resistance to temptations related to excessive smartphone use (Zhang et al., 2021). Ke et al. (2024) similarly reported a significant indirect association between PA and smartphone addiction involving self-esteem, suggesting that positive psychological resources may be statistically relevant to this relationship. Accordingly, PsyCap may represent one statistical pathway linking PA with MPD within the specified model.

The indirect association between PA and MPD through SC was also significant (standardized indirect association = −0.108, 95% CI [−0.157, −0.060]), supporting H3. Similar indirect links between physical activity and MPD through SC have been documented in earlier research (Li et al., 2022; Yang et al., 2019; Zhong et al., 2021). PA has been connected to prefrontal processes involved in decision-making, mood regulation, and behavioral control (Pan et al., 2023). It also requires students to follow schedules, adhere to plans, manage time, and regulate their behavior during exercise. These repeated self-regulatory demands may be associated with a greater capacity to inhibit impulsive behavior and manage the duration and intensity of smartphone use (Liu F. B. et al., 2022; Moore et al., 2022). Accordingly, SC may represent another statistical indirect pathway linking PA with MPD.

### 5.3 Serial indirect association through psychological capital and self-control

H4 was supported by the significant standardized serial indirect association between PA and MPD through PsyCap and SC (−0.036, 95% CI [−0.060, −0.014]), which accounted descriptively for 14.122% of the total association. This finding is consistent with earlier research that connected PsyCap with SC, PA with PsyCap, and SC with reduced MPD levels (Morrison and Pidgeon, 2017; Zhang et al., 2023; Zhong et al., 2021). This also corresponds to findings suggesting that several interrelated psychological factors may be involved in the association between PA and smartphone-related results (Wang J. F. et al., 2024). During PA, students may have opportunities to display their abilities, coordinate with peers, obtain social support, and build mutual trust (Bruner et al., 2014). Better athletic skills and more positive exercise experiences may be associated with greater self-efficacy, optimism, hope, and resilience, which together reflect higher PsyCap (Chen et al., 2022; Wei et al., 2024). Within the specified model, higher PsyCap was positively associated with SC, a pattern consistent with the proposed resource-to-regulation ordering in which broader psychological resources are linked to more proximal behavioral regulation. This pattern offers a more integrated theoretical account of the PA–MPD association by positioning PsyCap and SC as complementary psychological resources operating at different levels of self-regulation. The cross-sectional data support the proposed sequence but cannot establish temporal precedence or causality between PsyCap and SC.

## 6 Implications and limitations

### 6.1 Implications

Theoretically, this study extends previous research by considering PsyCap and SC simultaneously within the same model. The significant indirect associations through PsyCap and SC suggest that these constructs may represent complementary psychological pathways linking PA with MPD. PsyCap represents broader motivational and emotional resources, whereas SC reflects a more proximal capacity for behavioral regulation and impulse control. The significant serial indirect association was consistent with the theoretically specified ordering of PsyCap before SC. Thus, the study provides a more integrated account of the relationships among PA, PsyCap, SC, and MPD.

Practically, the findings may provide a useful reference for campus health promotion and student support programs addressing MPD. Universities may consider integrating regular PA opportunities with activities that foster PsyCap, such as goal setting, mastery experiences, positive feedback, and resilience building, as well as strategies that strengthen SC, such as self-monitoring, time management, and impulse regulation. Because the association between PA and MPD may vary across exercise types, program design may also take students’ exercise preferences, physical fitness, and appropriate exercise intensity into account (Yang et al., 2021). Overall, PsyCap and SC may represent potentially relevant psychological components to consider when developing PA-related and digital wellbeing initiatives for college students.

### 6.2 Limitations and future research directions

Several limitations warrant consideration. First, the use of convenience sampling, the uneven demographic composition of the sample, and the eligibility criterion requiring regular participation in PA for at least one academic semester may limit the generalizability of the findings, particularly to students with little or no PA experience. Future studies should recruit more balanced samples and include students with a broader range of PA participation levels. Second, neither causal inference nor the temporal ordering of PA, PsyCap, SC, and MPD can be established due to the cross-sectional design. To elucidate the direction and temporal sequence of these connections, longitudinal and experimental research is required. Third, answer bias, social desirability, and common method bias may have an impact on the simultaneous assessment of all variables using self-report measures. Moreover, the PSDQ Activity subscale reflects self-perceived participation in PA rather than objectively measured activity frequency, intensity, duration, or energy expenditure. Future studies may use behavioral tasks, multi-informant evaluations, or objective PA metrics. Finally, participants were recruited through classes at four universities, but class and university identifiers and the total number of participating classes were not retained. Consequently, the independence assumption and potential clustering within classes or institutions could not be evaluated. Future studies should retain relevant group identifiers and consider multilevel or cluster-adjusted analyses where appropriate.

## 7 Conclusion

This study investigated the relationship between PA and MPD and the indirect roles of PsyCap and SC. Although PA was negatively related to MPD, the direct PA–MPD link was no longer significant after adjustment for PsyCap, SC, and the covariates. PA was indirectly associated with lower MPD through PsyCap, SC, and the serial pathway involving PsyCap and SC. These findings suggest that PsyCap and SC may represent relevant psychological pathways in the association between PA and MPD. The findings clarify the relationships among PA, PsyCap, SC, and MPD and point to the value of future longitudinal and intervention studies.

## Data Availability

The raw data supporting the conclusions of this article will be made available by the authors, without undue reservation.
