# Supplementary material for: The association between physical activity and mobile phone dependence in college students: serial indirect associations through psychological capital and self-control
Source: Front Psychol. 2026 Oct 21;17:1911519. doi: 10.3389/fpsyg.2026.1911519 (PMC13731245; doi:10.3389/fpsyg.2026.1911519)
Supplement: Supplementary file 1 [file Data_Sheet_1.PDF]

Run MATRIX procedure:

***************** PROCESS Procedure for SPSS Version 4.2 *****************

Written by Andrew F. Hayes, Ph.D.   www.afhayes.com
Documentation available in Hayes (2022). www.guilford.com/p/hayes3

****
Model : 6
Y : MPD
X : PA
M1 : PsyCap
M2 : SC

Covariates:
Gender   Age   Grade

Sample
Size: 567

****
OUTCOME VARIABLE:
PsyCap

Model Summary

R	R-sq	MSE	F	df1	df2	p
.4270	.1823	148.4143	31.3259	4.0000	562.0000	.0000

Model

	coeff	se	t	p	LLCI	ULCI
constant	87.5717	3.6887	23.7408	.0000	80.3264	94.8169
PA	1.3844	.1278	10.8338	.0000	1.1334	1.6354
Gender	-.1572	1.0871	-.1446	.8851	-2.2925	1.9781
Age	.3047	1.4365	.2121	.8321	-2.5168	3.1262
Grade	.8717	.9540	.9137	.3613	-1.0022	2.7456

Standardized coefficients

	coeff
PA	.4240
Gender	-.0057
Age	.0097
Grade	.0421

****
OUTCOME VARIABLE:

SC

Model Summary

R	R-sq	MSE	F	df1	df2	p
.3102	.0962	21.3809	11.9486	5.0000	561.0000	.0000

Model

	coeff	se	t	p	LLCI	ULCI
constant	28.2240	1.9814	14.2445	.0000	24.3321	32.1159
PA	.2388	.0533	4.4781	.0000	.1341	.3435
PsyCap	.0576	.0160	3.5985	.0003	.0262	.0891
Gender	.3834	.4126	.9292	.3532	-.4271	1.1939
Age	-.1651	.5452	-.3028	.7622	-1.2361	.9059
Grade	-.5286	.3624	-1.4586	.1452	-1.2403	.1832

Standardized coefficients

	coeff
PA	.2028
PsyCap	.1597
Gender	.0386
Age	-.0146
Grade	-.0707

****

OUTCOME VARIABLE:

MPD

Model Summary

R	R-sq	MSE	F	df1	df2	p
.6081	.3698	7.2131	54.7752	6.0000	560.0000	.0000

Model

	coeff	se	t	p	LLCI	ULCI
constant	34.5776	1.3429	25.7475	.0000	31.9397	37.2154
PA	-.0352	.0315	-1.1152	.2653	-.0971	.0268
PsyCap	-.0405	.0094	-4.3065	.0000	-.0590	-.0220
SC	-.3694	.0245	-15.0631	.0000	-.4176	-.3212
Gender	-.2167	.2399	-.9035	.3667	-.6878	.2544
Age	-.0762	.3167	-.2406	.8100	-.6983	.5459
Grade	.1237	.2109	.5868	.5576	-.2905	.5379

Standardized coefficients

	coeff
PA	-.0429
PsyCap	-.1616

SC	-.5315
Gender	-.0314
Age	-.0097
Grade	.0238

************************* TOTAL EFFECT MODEL ***************************

OUTCOME VARIABLE:

MPD

Model Summary

R	R-sq	MSE	F	df1	df2	p
.2546	.0648	10.6662	9.7392	4.0000	562.0000	.0000

Model

	coeff	se	t	p	LLCI	ULCI
constant	18.7410	.9889	18.9521	.0000	16.7987	20.6833
PA	-.2089	.0343	-6.0981	.0000	-.2762	-.1416
Gender	-.3486	.2914	-1.1962	.2321	-.9211	.2238
Age	-.0340	.3851	-.0884	.9296	-.7904	.7224
Grade	.2651	.2558	1.0366	.3003	-.2372	.7675

Standardized coefficients

	coeff
PA	-.2552
Gender	-.0506
Age	-.0043
Grade	.0510

************** TOTAL, DIRECT, AND INDIRECT EFFECTS OF X ON Y **************

Total effect of X on Y

Effect	se	t	p	LLCI	ULCI	c_cs
-.2089	.0343	-6.0981	.0000	-.2762	-.1416	-.2552

Direct effect of X on Y

Effect	se	t	p	LLCI	ULCI	c'_cs
-.0352	.0315	-1.1152	.2653	-.0971	.0268	-.0429

Indirect effect(s) of X on Y:

	Effect	BootSE	BootLLCI	BootULCI
TOTAL	-.1738	.0257	-.2267	-.1240
Ind1	-.0561	.0145	-.0861	-.0283
Ind2	-.0882	.0208	-.1301	-.0486
Ind3	-.0295	.0099	-.0494	-.0109

Completely standardized indirect effect(s) of X on Y:

	Effect	BootSE	BootLLCI	BootULCI
TOTAL	-.2123	.0298	-.2709	-.1537
Ind1	-.0685	.0174	-.1036	-.0352
Ind2	-.1078	.0250	-.1568	-.0595
Ind3	-.0360	.0118	-.0598	-.0138

Indirect effect key:

Ind1 PA -> PsyCap -> MPD
Ind2 PA -> SC -> MPD
Ind3 PA -> PsyCap -> SC -> MPD

*********************** ANALYSIS NOTES AND ERRORS ************************

Level of confidence for all confidence intervals in output:
95.0000

Number of bootstrap samples for percentile bootstrap confidence intervals:
5000

------ END MATRIX -----
