# Supplementary material for: The association between physical activity and mobile phone dependence in college students: serial indirect associations through psychological capital and self-control
Source: Front Psychol. 2026 Oct 21;17:1911519. doi: 10.3389/fpsyg.2026.1911519 (PMC13731245; doi:10.3389/fpsyg.2026.1911519)
Supplement: Supplementary file 2 [file Data_Sheet_2.PDF]

Supplementary File S2

Analysis Syntax

SPSS PROCESS Model 6 syntax used for the serial indirect-association analysis.

The syntax specifies PA as X, PsyCap as M1, SC as M2, and MPD as Y, with gender, age, and grade included as covariates and 5,000 bootstrap resamples.

```
* Encoding: UTF-8.
PROCESS y=MPD
/x=PA
/m=PsyCap SC
/cov=Gender Age Grade
/model=6
/boot=5000
/total=1
/stand=1.
```

The syntax above is reproduced from the original .sps analysis file.
