# Supplementary material for: The association between physical activity and mobile phone dependence in college students: serial indirect associations through psychological capital and self-control
Source: Front Psychol. 2026 Oct 21;17:1911519. doi: 10.3389/fpsyg.2026.1911519 (PMC13731245; doi:10.3389/fpsyg.2026.1911519)
Supplement: Supplementary file 3 [file Table_1.DOCX]

**Supplementary Material**

**The Association Between Physical Activity and Mobile Phone Dependence in College Students: Serial Indirect Associations Through Psychological Capital and Self-Control**

**Supplementary Analysis Information**

The serial indirect-association model was estimated using the PROCESS macro for SPSS (Model 6). Physical activity (PA) was entered as X, psychological capital (PsyCap) as M1, self-control (SC) as M2, and mobile phone dependence (MPD) as Y. Gender, age, and grade were included as covariates. The analyses were conducted using the same final analytical sample of 567 participants used for the descriptive and correlation analyses. Indirect associations were evaluated using 5,000 percentile bootstrap resamples with 95% confidence intervals. An indirect association was considered statistically significant when its bootstrap confidence interval did not include zero. The main manuscript reports standardized regression coefficients and completely standardized indirect associations for ease of interpretation. The supplementary tables below additionally provide unstandardized coefficients, coefficients for all covariates, and complete total, direct, and indirect association estimates. The complete original PROCESS output is provided separately as Supplementary File S1.

**Supplementary Table S1**

Model Summary Statistics for the PROCESS Model 6 Regression Equations

| **Outcome Variable** | ***R*** | ***R²*** | ***MSE*** | ***F*** | ***df1*** | ***df2*** | ***p*** |
| --- | --- | --- | --- | --- | --- | --- | --- |
| PsyCap | 0.427 | 0.182 | 148.414 | 31.326 | 4 | 562 | < 0.001 |
| SC | 0.310 | 0.096 | 21.381 | 11.949 | 5 | 561 | < 0.001 |
| MPD | 0.608 | 0.370 | 7.213 | 54.775 | 6 | 560 | < 0.001 |
| MPD (total-association model) | 0.255 | 0.065 | 10.666 | 9.739 | 4 | 562 | < 0.001 |

*Note. N = 567. PsyCap = psychological capital; SC = self-control; MPD = mobile phone dependence; MSE = mean squared error.*

**Supplementary Table S2**

Complete Regression Coefficients for the PROCESS Model 6

| **Outcome** | **Predictor** | ***B*** | ***SE*** | ***β*** | ***t*** | ***p*** | **95% CI** |
| --- | --- | --- | --- | --- | --- | --- | --- |
| **PsyCap** | Constant | 87.572 | 3.689 |  | 23.741 | < 0.001 | [80.326, 94.817] |
|  | PA | 1.384 | 0.128 | 0.424 | 10.834 | < 0.001 | [1.133, 1.635] |
|  | Gender | −0.157 | 1.087 | −0.006 | −0.145 | 0.885 | [−2.293, 1.978] |
|  | Age | 0.305 | 1.437 | 0.010 | 0.212 | 0.832 | [−2.517, 3.126] |
|  | Grade | 0.872 | 0.954 | 0.042 | 0.914 | 0.361 | [−1.002, 2.746] |
| **SC** | Constant | 28.224 | 1.981 |  | 14.245 | < 0.001 | [24.332, 32.116] |
|  | PA | 0.239 | 0.053 | 0.203 | 4.478 | < 0.001 | [0.134, 0.344] |
|  | PsyCap | 0.058 | 0.016 | 0.160 | 3.599 | < 0.001 | [0.026, 0.089] |
|  | Gender | 0.383 | 0.413 | 0.039 | 0.929 | 0.353 | [−0.427, 1.194] |
|  | Age | −0.165 | 0.545 | −0.015 | −0.303 | 0.762 | [−1.236, 0.906] |
|  | Grade | −0.529 | 0.362 | −0.071 | −1.459 | 0.145 | [−1.240, 0.183] |
| **MPD** | Constant | 34.578 | 1.343 |  | 25.748 | < 0.001 | [31.940, 37.215] |
|  | PA | −0.035 | 0.032 | −0.043 | −1.115 | 0.265 | [−0.097, 0.027] |
|  | PsyCap | −0.041 | 0.009 | −0.162 | −4.307 | < 0.001 | [−0.059, −0.022] |
|  | SC | −0.369 | 0.025 | −0.532 | −15.063 | < 0.001 | [−0.418, −0.321] |
|  | Gender | −0.217 | 0.240 | −0.031 | −0.904 | 0.367 | [−0.688, 0.254] |
|  | Age | −0.076 | 0.317 | −0.010 | −0.241 | 0.810 | [−0.698, 0.546] |
|  | Grade | 0.124 | 0.211 | 0.024 | 0.587 | 0.558 | [−0.291, 0.538] |

*Note. N = 567. B = unstandardized regression coefficient; SE = standard error; β = standardized regression coefficient; CI = confidence interval. PA = physical activity; PsyCap = psychological capital; SC = self-control; MPD = mobile phone dependence.*

**Supplementary Table S3**

Complete Regression Coefficients for the Total-Association Model

| **Predictor** | ***B*** | ***SE*** | ***β*** | ***t*** | ***p*** | **95% CI** |
| --- | --- | --- | --- | --- | --- | --- |
| Constant | 18.741 | 0.989 |  | 18.952 | < 0.001 | [16.799, 20.683] |
| PA | −0.209 | 0.034 | −0.255 | −6.098 | < 0.001 | [−0.276, −0.142] |
| Gender | −0.349 | 0.291 | −0.051 | −1.196 | 0.232 | [−0.921, 0.224] |
| Age | −0.034 | 0.385 | −0.004 | −0.088 | 0.930 | [−0.790, 0.722] |
| Grade | 0.265 | 0.256 | 0.051 | 1.037 | 0.300 | [−0.237, 0.768] |

*Model summary: R = 0.255, R² = 0.065, MSE = 10.666, F (4, 562) = 9.739, p < 0.001.*

*Note. N = 567. PA = physical activity; B = unstandardized regression coefficient; SE = standard error; β = standardized regression coefficient; CI = confidence interval.*

**Supplementary Table S4**

Total, Direct, and Indirect Associations Between PA and MPD

| **Pathway** | ***B*** | **SE /Boot SE** | **95% CI** | **Completely Standardized Estimate** | **Standardized Boot SE** | **Standardized Bootstrap 95% CI** |
| --- | --- | --- | --- | --- | --- | --- |
| Total association | −0.209 | 0.034 | [−0.276, −0.142] | −0.255 |  |  |
| Direct association | −0.035 | 0.032 | [−0.097, 0.027] | −0.043 |  |  |
| Total indirect association | −0.174 | 0.026 | [−0.227, −0.124] | −0.212 | 0.030 | [−0.271, −0.154] |
| PA → PsyCap → MPD | −0.056 | 0.015 | [−0.086, −0.028] | −0.069 | 0.017 | [−0.104, −0.035] |
| PA → SC → MPD | −0.088 | 0.021 | [−0.130, −0.049] | −0.108 | 0.025 | [−0.157, −0.060] |
| PA → PsyCap → SC → MPD | −0.030 | 0.010 | [−0.049, −0.011] | −0.036 | 0.012 | [−0.060, −0.014] |

***Note.*** *N = 567. B = unstandardized estimate; SE = standard error; Boot SE = bootstrap standard error; CI = confidence interval. SE is reported for the total and direct associations, whereas Boot SE is reported for the indirect associations. Bootstrap confidence intervals for indirect associations were based on 5,000 percentile bootstrap resamples. PA = physical activity; PsyCap = psychological capital; SC = self-control; MPD = mobile phone dependence.*

**Supplementary Table S5**

Relative Proportions of the Indirect Associations

| **Indirect Association** | **Relative Proportion (%)** |
| --- | --- |
| Total indirect association | 83.198 |
| PA → PsyCap → MPD | 26.855 |
| PA → SC → MPD | 42.221 |
| PA → PsyCap → SC → MPD | 14.122 |

***Note.*** *The relative proportion was calculated by dividing each unstandardized indirect association by the unstandardized total association and multiplying by 100%. The percentages are presented descriptively and should not be interpreted as statistical comparisons among the indirect pathways.*

**Supplementary Table S6**

Standardized Factor Loadings for the Confirmatory Factor Analysis Models

| **Construct** | **Factor** | **Item** | **Standardized_Loading** | ***p*_value** |
| --- | --- | --- | --- | --- |
| PA | Physical Activity | PA1 | 0.733 | < 0.001 |
| PA | Physical Activity | PA2 | 0.731 | < 0.001 |
| PA | Physical Activity | PA3 | 0.782 | < 0.001 |
| PA | Physical Activity | PA4 | 0.756 | < 0.001 |
| PA | Physical Activity | PA5 | 0.771 | < 0.001 |
| PA | Physical Activity | PA6 | 0.781 | < 0.001 |
| PsyCap | Self-efficacy | PC1 | 0.540 | < 0.001 |
| PsyCap | Self-efficacy | PC3 | 0.554 | < 0.001 |
| PsyCap | Self-efficacy | PC5 | 0.538 | < 0.001 |
| PsyCap | Self-efficacy | PC7 | 0.501 | < 0.001 |
| PsyCap | Self-efficacy | PC9 | 0.540 | < 0.001 |
| PsyCap | Self-efficacy | PC11 | 0.536 | < 0.001 |
| PsyCap | Self-efficacy | PC13 | 0.533 | < 0.001 |
| PsyCap | Resilience | PC2 | 0.518 | < 0.001 |
| PsyCap | Resilience | PC4 | 0.549 | < 0.001 |
| PsyCap | Resilience | PC6 | 0.505 | < 0.001 |
| PsyCap | Resilience | PC8 | 0.505 | < 0.001 |
| PsyCap | Resilience | PC10 | 0.509 | < 0.001 |
| PsyCap | Resilience | PC12 | 0.571 | < 0.001 |
| PsyCap | Resilience | PC14 | 0.503 | < 0.001 |
| PsyCap | Hope | PC15 | 0.525 | < 0.001 |
| PsyCap | Hope | PC17 | 0.569 | < 0.001 |
| PsyCap | Hope | PC19 | 0.603 | < 0.001 |
| PsyCap | Hope | PC21 | 0.603 | < 0.001 |
| PsyCap | Hope | PC23 | 0.573 | < 0.001 |
| PsyCap | Hope | PC25 | 0.595 | < 0.001 |
| PsyCap | Optimism | PC16 | 0.618 | < 0.001 |
| PsyCap | Optimism | PC18 | 0.646 | < 0.001 |
| PsyCap | Optimism | PC20 | 0.629 | < 0.001 |
| PsyCap | Optimism | PC22 | 0.642 | < 0.001 |
| PsyCap | Optimism | PC24 | 0.638 | < 0.001 |
| PsyCap | Optimism | PC26 | 0.589 | < 0.001 |
| SC | Self-Control | SC1 | 0.514 | < 0.001 |
| SC | Self-Control | SC2 | 0.554 | < 0.001 |
| SC | Self-Control | SC3 | 0.583 | < 0.001 |
| SC | Self-Control | SC4 | 0.626 | < 0.001 |
| SC | Self-Control | SC5 | 0.606 | < 0.001 |
| SC | Self-Control | SC6 | 0.626 | < 0.001 |
| SC | Self-Control | SC7 | 0.666 | < 0.001 |
| SC | Self-Control | SC8 | 0.662 | < 0.001 |
| SC | Self-Control | SC9 | 0.690 | < 0.001 |
| SC | Self-Control | SC10 | 0.626 | < 0.001 |
| SC | Self-Control | SC11 | 0.565 | < 0.001 |
| SC | Self-Control | SC12 | 0.563 | < 0.001 |
| SC | Self-Control | SC13 | 0.547 | < 0.001 |
| MPD | Mobile Phone Dependence | MPD1 | 0.815 | < 0.001 |
| MPD | Mobile Phone Dependence | MPD2 | 0.814 | < 0.001 |
| MPD | Mobile Phone Dependence | MPD3 | 0.818 | < 0.001 |
| MPD | Mobile Phone Dependence | MPD4 | 0.797 | < 0.001 |
| MPD | Mobile Phone Dependence | MPD5 | 0.761 | < 0.001 |

***Note.*** *Standardized factor loadings were obtained from confirmatory factor analyses estimated using WLSMV in R with the lavaan package. PA, SC, and MPD were specified as single-factor models, whereas PsyCap was specified as a four-factor model comprising self-efficacy, resilience, hope, and optimism. No items were deleted, and no correlated residuals or other post hoc model modifications were introduced. PA = physical activity; PsyCap = psychological capital; SC = self-control; MPD = mobile phone dependence.*

**Supplementary Table S7**

Fit Indices for the Confirmatory Factor Analysis Models

| **Construct** | ***χ²*** | ***df*** | ***χ²/df*** | ***CFI*** | ***TLI*** | ***RMSEA*** | **95% CI for RMSEA** | ***SRMR*** |
| --- | --- | --- | --- | --- | --- | --- | --- | --- |
| PA | 13.480 | 9 | 1.498 | 0.999 | 0.998 | 0.030 | [0.000, 0.060] | 0.015 |
| PsyCap | 364.051 | 293 | 1.242 | 0.975 | 0.972 | 0.021 | [0.013, 0.027] | 0.038 |
| SC | 170.293 | 65 | 2.620 | 0.943 | 0.932 | 0.053 | [0.044, 0.063] | 0.041 |
| MPD | 2.976 | 5 | 0.595 | 1.000 | 1.000¹ | 0.000 | [0.000, 0.044] | 0.007 |

***Note.*** *CFA = confirmatory factor analysis; CFI = comparative fit index; TLI = Tucker–Lewis index; RMSEA = root mean square error of approximation; SRMR = standardized root mean square residual. Models were estimated using WLSMV with the Likert-type items treated as ordered categorical indicators. No correlated residuals, item deletions, or other post hoc modifications were introduced. ¹The software-produced TLI for the MPD model was 1.001; it is reported as 1.000 for presentation because incremental fit indices are conventionally bounded at 1.00.*

**Supplementary File S1**

Complete PROCESS Output

The complete original PROCESS Model 6 output generated in SPSS is provided separately as:

Supplementary_File_S1_PROCESS_Output.pdf

The output includes the complete model summaries, unstandardized and standardized coefficients for the focal variables and covariates, the total-association model, the direct association, the total indirect association, and the three specific indirect associations. Bootstrap standard errors and 95% bootstrap confidence intervals for the indirect associations were based on 5,000 resamples.

**Supplementary File S2**

Analysis Syntax

The SPSS PROCESS syntax used to estimate the serial indirect-association model is provided separately as:

Supplementary_File_S2_Analysis_Syntax.pdf

The syntax specifies PROCESS Model 6 with PA entered as X, PsyCap as M1, SC as M2, and MPD as Y, with gender, age, and grade included as covariates and 5,000 bootstrap resamples used to evaluate the indirect associations.
